# Engineering a DNA origami mediated multicolour quantum dot platform for sub-diffraction spectral separation imaging†

**DOI:** 10.1039/d2ra04316e

**Published:** 2022-08-22

**Authors:** Da Huang, Lucy Haddad, Fahmida Rahman, Matteo Palma, Andrei Sapelkin

**Affiliations:** Department of Chemistry, Queen Mary University of London London E1 4NS UK; Department of Physics and Astronomy, Queen Mary University of London London E1 4NS UK a.sapelkin@qmul.ac.uk

## Abstract

The validation of super-resolution optical imaging techniques requires well-defined reference samples that can be used repeatedly and reliably as model standards. Here, we engineer a DNA origami scaffold-mediated multicolour quantum dot hybrid nanostructure and test it using a recently proposed Quantum Dot-based spectral separation technique. We show that multivalent DNA structures offer a robust and precise nanoscale quantum dot placement scaffold, while the spectral resolution method provides relatively simple and fast image acquisition capabilities using any standard confocal or fluorescence microscope capable of spectral signal separation and a single excitation laser wavelength.

## Abstract 2

The validation of super-resolution optical imaging techniques requires well-defined reference samples that can be used repeatedly and reliably as model standards.

## Graphical abstract

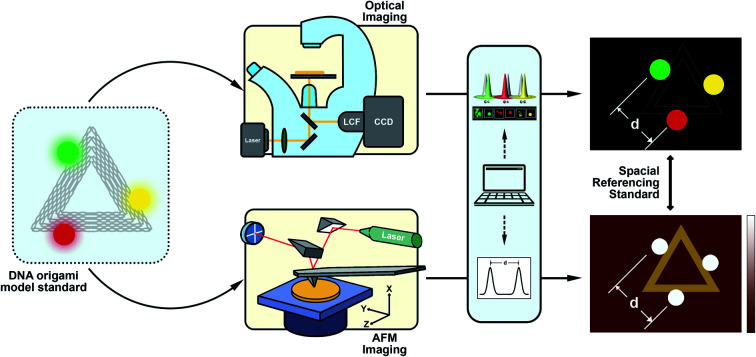

## Introduction

The ability to organize the assembly of individual molecules with nanoscale spatial control and fast imaging below the optical diffraction limit is of paramount importance in many fields, from optoelectronics to biotechnology. Applications range from engineering precise nanocavity emission arrays for highly sensitive sensing, to visualizing nanoscale deformation of materials, and mapping molecule spatial diffusion in live cells.^1–3^ In this regard, multivalent nanoarchitectures for probing and mapping spatial localized information of targets at the nanoscale, and the methods of imaging these supramolecular structures and their subdomains, are highly demanded.^4–8^ Additionally, a universal and robust platform is also essential for the validation of super-resolution imaging methods that demand comprehensive and accurate characterization by using appropriate reference standards.

Over the years, several super-resolution imaging methods have been developed^9–11^ that are based on fluorophore localisation including Stimulated Emission Depletion (STED),^12–14^ Photoactivation Localization Microscopy (PALM) and Stochastic Optical Reconstruction Microscopy (STORM).^15,16^ Imaging resolution using these methods has been improved down to molecular dimensions offering new insight into biological structures. However, these methods require high light intensity levels which build up toxic products in cells,^14^ and/or need an accumulation of thousands of images to establish the quantified means of intermolecular spacing.^17^ In addition to the imaging instrument configurations, the source of the light emission, such as fluorescent dyes, is also crucial in super-resolution imaging. It has been shown that a variety of approaches can be used that rely on photophysical properties of fluorophores such as photo-switching, photobleaching, blinking, DNA-PAINT, fluorescence lifetime and spectral differences.^18–27^ In some methods, organic fluorophores can achieve high precision localization but require a specialized microscope set up with the number of excitation wavelengths (to match the excitation spectra of the molecular fluorophores).^28^

It was recognised early on that standardisation of a variety super-resolution imaging methods requires a suitable standards that can be used to characterise an optical systems and these have been developed^29–31^ using the DNA-origami approach. In the vast majority of cases, it is organic dies dyes that are used as fluorescent markers. However, there are significant limits to the organic dye stability and longevity. The DNA-PAINT technique does address the problem of fluorophore stability to some extent, however, is rather time-consuming. Frequency-multiplexed DNA-PAINT approach does address some of the issues, but at an expense of experimental complexity as multiple laser excitation wavelength are required.^19^ In contrast, quantum dots (QDs) offer several advantages such as long shelf life, efficient broadband light harvesting capability, tuneable emission spectra controlled by particle size, solution processability and are also highly resistant to photobleaching.^32–35^ Furthermore, unlike molecular-based emitters, QDs can offer significant gains in photon flux albeit at the size cost. Thus, stable model reference QD-based nanostructures would bring significant benefits for validating the super-resolution imaging method.^36–38^ In this regard, a preferred artificial model structure will require both nanoscale spatial resolution as well as stoichiometric control for the arrangement of a pre-defined number of markers.

To utilize the advantages of QDs and to improve the efficiency of acquiring super-resolution imaging, we have previously proposed to use a spectral rather than a temporal signal separation approach.^34^ Combining this spectral separation approach with single-molecule localization microscopy methods, a random mixture of different-sized QDs in different emission channels can be localized and identified.^39,40^ However, just as for the other super-resolution methods, spectral separation approach requires the characterization of the optical system. The ideal platform to test and assess this Quantum Dot-based optical spectral separation (QDOSS) would require precise nanoscale control and multivalent single-molecule stoichiometric resolution.

In this context, DNA self-assembled nanostructures (DNA origami) have already been shown to be ideal for the creation of multivalent nanostructures and reference model standards.^29–31^ DNA can be used as building block, folded into predetermined shapes based on base-parings of the designed sequences, and further allow positioning of molecular moieties, including fluorophores, at precise location with a theoretical spatial resolution down to 6 nm in plane and 30 nm out of plane,^41–43^ allowing for highly complex arrangements towards a multivalent model standard.^44–48^

In this work, we engineered a triangular DNA origami nanoscaffold for the controlled assembly of distinct multicoloured QDs arranged in a multivalent model reference standard. We used these to test the QDOSS approach to acquire rapid and easily accessible super-resolution images. The proposed method can be relatively easily implemented from standard off-the shelf components or using any microscope capable of or adapted to spectral signal separation with a single excitation laser wavelength. Besides, QDOSS approach allows for fast verification of assembled DNA-QD hybrid structures.

## Results and discussion

**Fig. 1:**
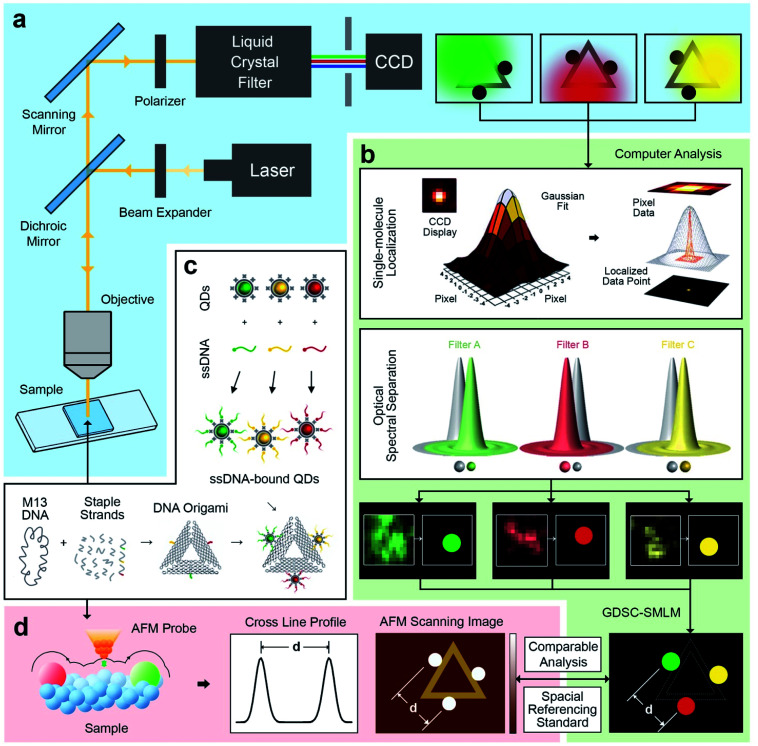
Schematic of DNA origami mediated quantum Dot-based optical spectral separation for sub-diffraction super-resolution imaging. (a) Epifluorescence system for optical imaging of DNA origami with multicolour quantum dots, with a liquid crystal filter for separating the light emitted from the sample into specific wavelengths. (b) Computer aided image processing with 2D Gaussian peak fitting localization and optical spectral separation with a localization accuracy of 5 ± 1 nm. The spatial analysis results of multicolour quantum dots can be compared with AFM evaluation and utilized as special referencing standard for various sub-diffraction imaging. (c) Synthesis of triangular DNA origami with spatial controlled binding of multicolour quantum dots *via* specific sticky end sequence recognizing strategy. (d) AFM characterization of multivalent DNA origami. The analysis of cross line profile and scanned image provides the spatial information on quantum dots.

An illustration of the multivalent DNA standard referenced Quantum Dot-based optical spectral separation platform is shown in Fig. 1. Generally, any microscopy system capable of spectral signal separation (*e.g.*, Leica TCS series) can be used for super-resolution imaging of the DNA-QD hybrid system. Here we demonstrate that super-resolution images can be obtained using a single laser excitation wavelength and a custom-built fluorescence microscope (Fig. 1a). The liquid crystal filter (Thorlabs Kurios-WB1) electronically synchronized with a CCD detector (Andor Clara E) has been used for signal separation in this case. By appropriately setting the wavelength of the liquid crystal filter, the light emitted by the entire reference standard (multicolour QDs assembled DNA origami) can be divided into three wavelengths corresponding to the peak emission of the each of the QDs (525 nm, 605 nm, and 655 nm). In this arrangement, the light emitted by individual QDs is recorded at each filter setting and three images are individually processed using the Genome Damage and Stability Centre (GDSC) Single Molecule Light Microscopy (SMLM) ImageJ Plugin,^49^ providing the super resolution image reconstruction (Fig. 1b). This arrangement allows for the localization of individual QDs, thereby greatly increasing the resolution of the optical system.^50^

To further test this methodology and to calibrate the system's precision, we synthesized DNA origami reference standards with accurate allocation control of multicoloured QDs (Fig. 1c), and validated the actual spatial control of QDs using atomic force microscopy (AFM) (Fig. 1d). The DNA origami standards can be synthesized *via* self-assembly based on sequences design. For the purpose of spectral separation of multicolour QDs, a multivalency QDs modification strategy was developed. Different QDs can be precisely allocated in the certain position on DNA origami scaffold and characterized *via* AFM. Because of the QDs size (about 10–20 nm), their actual position on the DNA nanostructures can be clearly revealed under AFM. A cross-section profiling can be used to measure the distance between each QD and then compare that with the super-resolved data from the optical method. Based on the DNA origami design, distinct QDs were separated from each other by around 70 nm, which can be further validated by comparison of standard-AFM with standard-QDOSS. Hence the ability to achieve sub-diffraction imaging utilizing our spectral separation approach can be demonstrated.

### Multivalent DNA origami

**Fig. 2:**
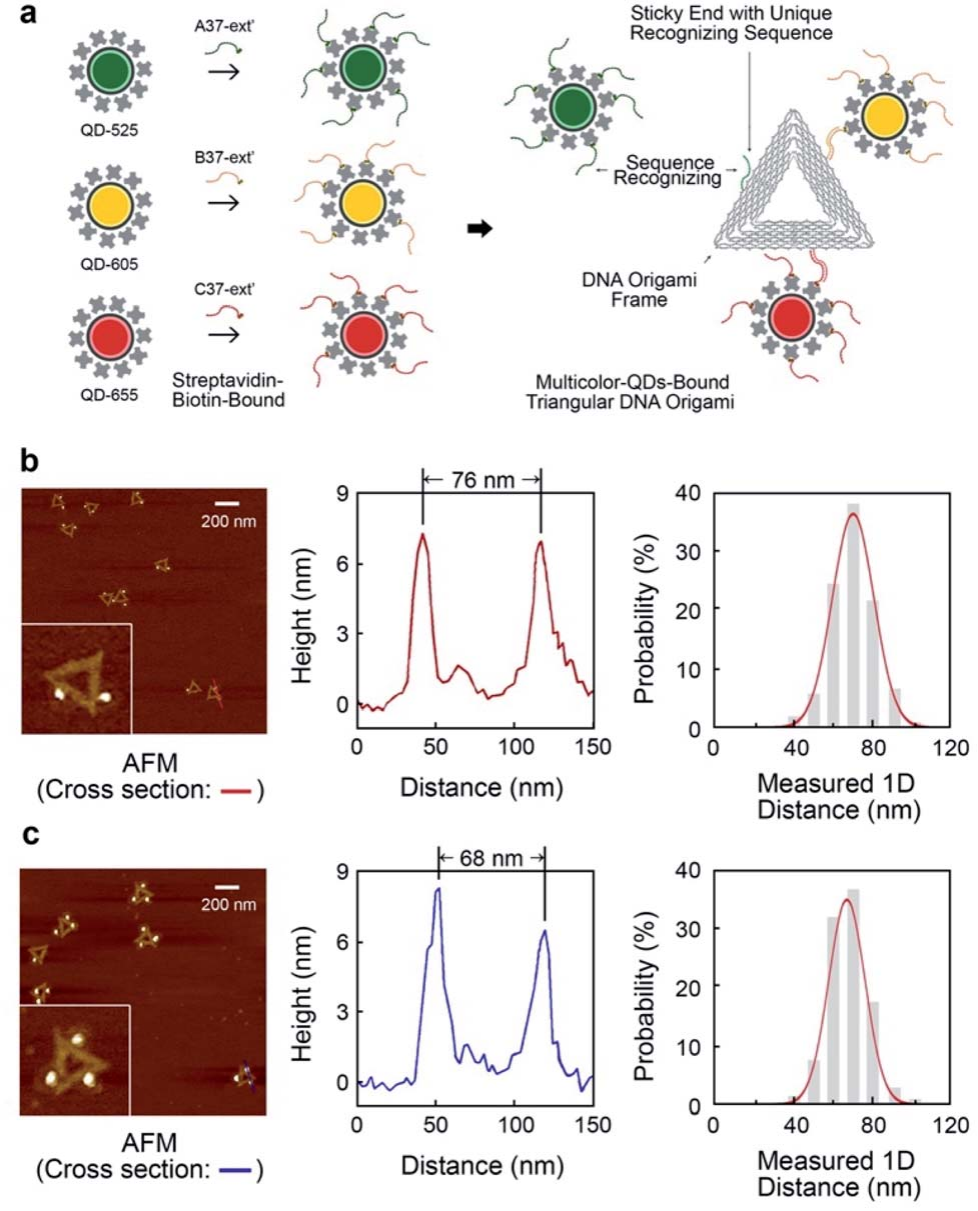
Multivalent triangular DNA origami with multicolour quantum dots assembly. (a) Schematic showing synthesizing multivalent optical moieties on single DNA origami *via* unique sequence recognizing the sticky ends on each side of DNA origami and the ssDNA bound to QDs. QD-525, QD-605 and QD-655 were attached in the 3-QDs-bound system. The certain position of chosen quantum dot can be spatially controlled. (b) The characterization of 2-QDs-Bound DNA origami. AFM images with cross-section reveal the distances between quantum dots on a single DNA origami. Distribution of inter-QDs distances shows the statistics of spatially controlled quantum dots on DNA origami. (c) The characterization of 3-QDs-bound DNA origami with AFM scanning, cross-section analysis and QDs spatial distribution.

The triangular DNA origami shown in Fig. 2 (side length of 120 nm) was used as the model standard, and modified with 2 or 3 different distinct types of QDs. The desired DNA origami nanostructure could not present a single conjugation chemistry: *e.g.*, using the same streptavidin conjugate coating, *via* direct biotin linkage approach, would lead to a non-controllable of the type of QD on the origami.

In order to develop multivalent QD assembly, a two-steps approach was carried out consisting of a biotinylated ssDNA-QD conjugation and DNA hybridization between DNA functionalized QD and the sticky end on origami (Fig. 2a). Once a QD is conjugated with a specific biotinylated ssDNA strand, this QD can be assembled to DNA origami specifically designed with a sticky end complementary to the biotinylated ssDNA on the QD, *via* DNA hybridization. As a result, unique sticky ends would only hybridize to the complementary biotinylated ssDNA which is bound to the corresponding QD of interest. By this strategy, selected numbers and type of QDs can assemble on origami utilizing the design of different sequences of sticky ends and complementary biotinylated single strands. One QD is coated with 10 to 14 streptavidin, which means DNA modified QDs have more than one ssDNA. This increases the efficiency of QD binding to the sticky ends present on the DNA origami.

Triangular DNA origami were programmed for the assembly of three different streptavidin conjugated QDs with a maximum emission at 525 nm, 605 nm and 655 nm (in the case of two QDs modification, 585 nm and 705 nm were selected). In this arrangement, the distance between the different QDs is approximately 70 nm, by design. Atomic force microscopy (AFM) imaging was employed to verify the attachment of QDs to the DNA origami *via* cross-section profiling and measured distancing (Fig. 2b and c) and their size (Fig. S4†). From AFM imaging, it was found that the distance between the QDs varies between 60 nm to 90 nm, due to the flexibility of the DNA strands anchoring the QDs to the Origami. The mean observed distances are 73 nm and 69 nm, for 2-QDs-bound and 3-QDs-bound DNA origami standards respectively. Size analysis (see Fig. S4†) indicates that we did achieve the required targeted attachment (this was further corroborated by the optical super-resolution imaging shown below). These reference standards were then employed for the comparison with the super-resolution images obtained using spectral signal separation.

### QDOSS imaging

The same multivalent DNA standard samples were then used to collect images using both a custom-built system and a conventional confocal microscope capable of spectroscopic signal separation (Leica TCS SP2). Since 2 or 3 different QDs were attached to DNA origami, distinct emission ranges were set for the imaging of each type of QDs. Whereas the emission ranges were 570–630 nm for QD585, 650–800 nm for QD705 in the 2-QDs-bound system, we set the emission ranges to 490–560 nm for QD525, 570–620 nm for QD585, and 635–700 nm for QD655 in the 3-QDs-bound system. With the custom-build system, the bandpass of the liquid crystal filter was set to the central emission wavelength of the corresponding QDs.

**Fig. 3:**
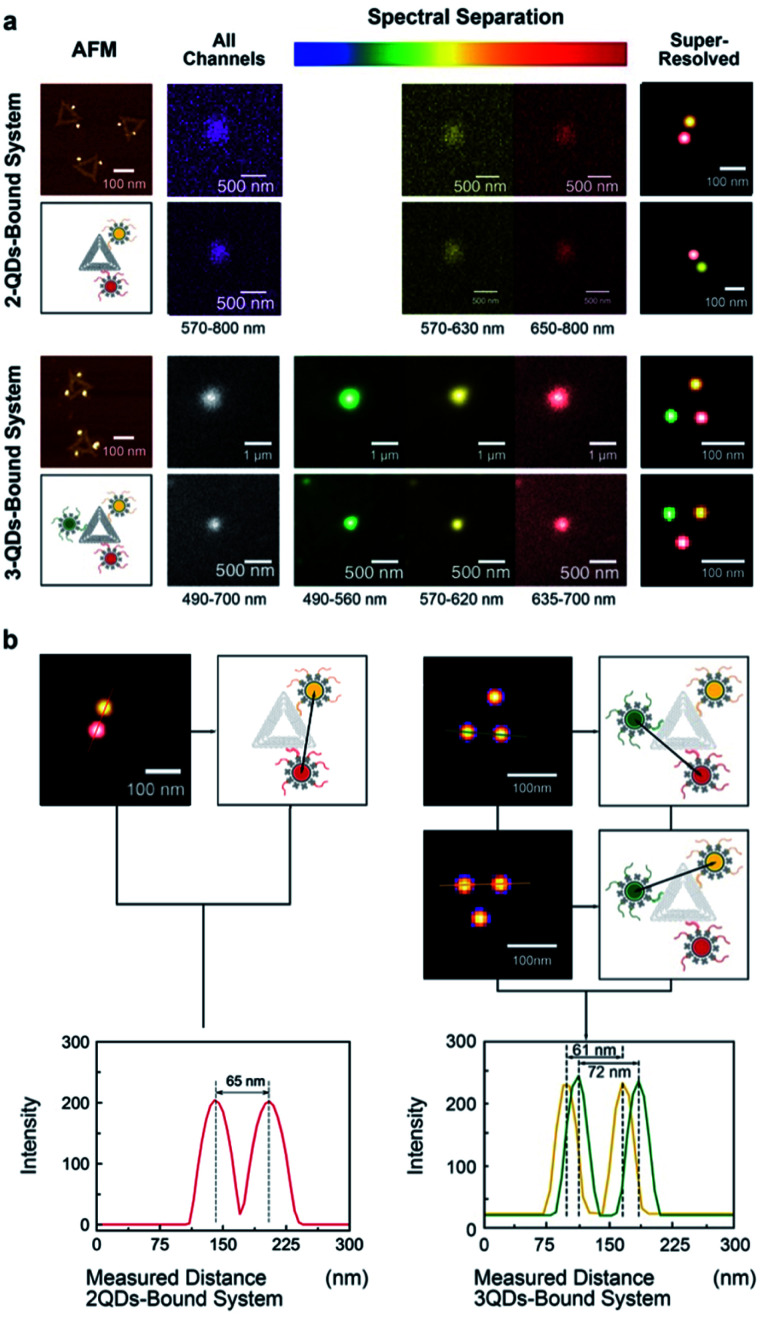
Super-resolution imaging of multicolour QDs attached to triangular DNA Origami. (a) AFM, raw optical images, and reconstructed images of 2-QDs and 3-QDs-bound systems. Fluorescent signal spots were spectrally separated, and localization images were reconstructed for each system. Sums of all images (grey) represent the images without spectral separation (the emission wavelength ranges from 570 nm to 800 nm in 2-QDs system and 490 nm to 700 nm in 3-QDs system). Spectrally separated images and the localizations in the reconstructed images were recoloured as green for QD-525, yellow for QD-605 and red for QD-655. (b) The cross-section characterization of 2-QDs-Bound and 3-QDs-Bound DNA origami standards after spectral reconstruction. Distances between quantum dots on a single DNA origami were measured based on super-resolved images.

All image frames were processed using an ImageJ peak fitting and localization plugin, GDSC SMLM, to obtain SR images (Fig. 3). The software identifies the local maxima of intensity in the image and then fits a 2D Gaussian function.^49^ The super-resolution cross-section characterization was carried out on both of 2-QDs-Bound and 3-QDs-Bound DNA origami standards after the signal reconstruction. Distances between quantum dots modified on a single DNA origami were measured based on super-resolved images. An average distance of 71 nm was revealed corresponding to the 2-QDs-Bound DNA origami standard, and distances between 61 nm and 72 nm were revealed corresponding to 3-QDs-Bound DNA origami standard. The average distance between the localizations seen in the reconstructed images was found as 75 ± 15 nm. Comparison of the AFM and the reconstructed SR images shows consistency in the patterns and distances of QDs attached to DNA origami, thus confirming an optical resolution down to at least 60 nm (with localization precision between 2 and 11 nm, depending on the signal-to-noise ratio, see attached tables with data analysis information), *i.e.*, well below the diffraction limit (Fig. 3a; large area diffraction-limited and super-resolution fluorescence images of multicolour QDs that include multiple triangular origami are shown in Fig. S8†). We found the super-resolved optical images to be consistent with the AFM results for the DNA origami reference standards, while revealing the colour combination that cannot be observed under AFM (Fig. 3b). The distance measurements the AFM characterization results within the experimental errors (see Fig. 2) and validate the QDOSS imaging method based on the multicolour QDs modified DNA origami standard.

## Conclusions

In summary, we engineered a novel multivalent DNA origami reference standard where multicolour QDs are arranged with precise spatial control. We have demonstrated that these hybrid structures can be used as super-resolution reference standards in our QDOSS method using a mixture of QDs with distinctive well-separated peak emission wavelengths using standard epifluorescence or confocal fluorescence microscope capable of spectral signal separation and equipped with a single wavelength laser. Additionally, thanks to the sufficient photon flux from QDs, the laser power requirements are significantly reduced with our QDOSS approach compared to other SR methods, with intensities as low as <30 W cm^−2^ used in this study. Importantly, by designing appropriate filters/detectors/QDs arrangements, the QDOSS methodology can be extended to simultaneous multi-wavelength data collection (for instance using several detectors, or multiple images on a single detector) and can in principle be used for sub-millisecond acquisition times, thus providing a clear potential for long-term live super-resolution co-localization and imaging. Moreover, the approach presented here is for general applicability to other fluorescent materials where the nature of the emission is due to exciton quantum confinement (including carbon dots or fluorescent diamonds).

Currently, the key drawback of the QDOSS approach is the requirement for neighbouring QDs to be of different peak emission wavelength with no (or minimal) overlap, hence the limited selection of emission wavelengths in our proof-of-principle experiments. Thus, today the proposed technique is perhaps much more suitable for co-localization below the diffraction limit, rather than for general super-resolution imaging. The route towards the latter is through utilizing a wider selection of QDs with narrow emission wavelengths, and by employing labelling strategies that inhibit close proximity of QDs with similar emission profiles. Ultimately, the approach is limited by the intrinsic QDs emission peak broadening and peak overlap, that would require additional post-processing (*e.g.*, deconvolution or multi-parameter fitting).

## Experimental section

### Synthesis of triangular DNA origami nanostructures

DNA origami is synthesized *via* mixing M13mp18 (5 nM) and staple strands (50 nM) in 50 μL 1 × TAE buffer with 12.5 mM Mg^2+^ (see DNA sequences in ESI†). M13mp18 is a bacteria phage vector strand with 7249 bases long. An appropriate quantity of ions, such as magnesium here or sodium in the DNA hybridization approach, are demanded to equilibrate the electrostatic repulsion between highly negatively charged DNAs molecules. An amount of 12.5 mM Mg^2+^ was chosen based on balancing the increase of the yield and reduction of the aggregation. The mixture solution is heated to 94 °C to completely dihybridise all of dsDNA. Temperature step controlled cooled down approach is carried out in a PCR machine. From 94 °C to 65 °C, cooling rate is about at 0.3 °C per minute. A cooling rate of 0.1 °C per minute is employed from 65 °C to room temperature. The self-assembled DNA origami is purified with Millipore Amicon Ultra 100 kDa spin columns in a centrifuge at 13 000 rpm for 2 minutes, 3 times, to get rid of access staple strands. Residues in the spin column is adjusted to a concentration around 20 nM by regulating the volume to about 50 μL. A NanoDrop Spectrophotometer is used to detect the rough concentration of DNA origami products based on the constant of a molecular weight of 330 g mol^−1^ per base and an extinction coefficient = 33 mg mL^−1^ for *A*_260_ = 1. The actual result is normally close to the estimated numbers.

### Quantum dots modification on DNA origami

Three different QDs were assembled on DNA origami using the sequence recognition directed by ssDNA complimentary to sticky-ends. The position of QD on a single origami depends on the location of complementary sticky-ends that origami has. Three positions were chosen in this study (namely at A37, B37, and C37 of a triangular DNA origami, see ESI†). Commercially streptavidin conjugated QDs (Qdot® 525 Streptavidin Conjugate, Qdot® 605 Streptavidin Conjugate, Qdot® 655 Streptavidin Conjugate, Invitrogen) was attached with corresponding recognition biotinylated ssDNA guiding strands (A37-ext′, B37-ext′ and C37-ext′) *via* incubating under room temperature for 30 minutes with shacking. The ratio of ssDNA towards QD was 50 : 1 that performs full coverage of the streptavidin binding sites. The access ssDNA was removed after conjugation *via* molecule weight cut-off spin filter. The unique QD was assembled with correspondence recognition ssDNA separately, but combined together when carrying out the assemble on DNA origami. The multicolour QD-ssDNA conjugation mixture was added in origami solution and incubated with shacking over 2 hours. The calculated ratio of QD-ssDNA conjugation to unique sticky-ends was 1 to 1 in order to avoid access QDs.

### Deposition of DNA on substrates

DNA origami was first diluted 20 times in Tris buffer (5 mM; pH 8.2) with 30 mM Mg^2+^. 60 μL of the DNA origami solution was cast on the substrate and placed in a 6-wells plate with moist Kimwipe. The sample was incubated for 90 minutes on a shaker. The sample was then washed with Tris buffer (5 mM; pH 8.2) with 30 mM Mg^2+^ (60 μL × 8). A 0.01% solution of carboxyethylsilane in the same Tris buffer was washed in with (60 μL × 8), and the sample was incubated for 2 minutes on a shaker. The buffer was then exchanged for MOPS buffer (10 mM; pH 8.1) with 30 mM Mg^2+^ (60 μL × 8). An equal volume of EDC (1-ethyl-3-(3-dimethylaminopropyl) carbodiimide; 50 mM) and NHS (*N*-hydroxysulfosuccinimide; 100 mM) in the MOPS buffer was added to the sample's volume and the sample was incubated for 10 minutes on a shaker. The sample was washed with the MOPS buffer, then rinsed with DPBS with 125 mM NaCl to remove any uncovalently bound structures, and subsequently rinsed with water. The samples were checked under AFM.

### AFM characterization of DNA origami

AFM was used to image the DNA origami structures. DNA origami is cast on either silicon dioxide, glass or mica surfaces for imaging. The DNA origami solution is diluted by TAE buffer with 30 mM Mg^2+^ to around 1 nM in order to get a good separation of the DNA nanostructures once immobilized on surface. Magnesium is required in the procedure as an ion charged bridge, immobilizing DNA origami to the substrate surfaces. Mica samples were cleaved twice by solid scotch tape immediately prior casting. 5 μL of diluted DNA origami solution was directly deposited on freshly cleaned mica and left to adsorb on the surface for 2 min. Subsequently, the substrate was washed by distilled water to remove non-absorbed origami and then blown dry by compressed air. ScanAsyst-Air tips with 0.4 N m^−1^ spring constant were used to scan the sample by AFM under ScanAsyst™ Mode. A resolution of 512 pixels per line with 1 Hz scan rate was chosen for appropriate imaging of the DNA nanostructure.

### QDOSS imaging

The custom-built imaging system consists of Nikon APO TIRF 100 × 1.49 NA oil immersion microscope objective, Kimmon He–Cd 442 nm laser used for excitation, Thorlabs Kurios-WB1 liquid crystal filter and Andor Clara E CCD detector (pixel size 6.45 μm). The laser beam was expanded and then focused on the back focal plane of the objective to operate the system in the epi-fluorescence mode. The laser light was rejected by a 442 nm notch filter (Kaiser Optical Systems holographic supernotch filter) and emission signal was passed through a linear polarizer (Thorlabs LPVISC100-MP2) aligned with the filter plane. The detector gain and read noise were calculated using Genome Damage and Stability Centre Single Molecule Localization Microscopy plugin from the appropriate images.^49^ The sample positioning was carried out using Prior H101RNSW XY stage with Prior H127PSIV controller. The fine focusing along *z*-axis was achieved using Piezosystem Jena MIPOS 100 PL SG objective lens piezo positioning system with NV40/1 CLE piezo controller. All data were collected from the field of view of no more than 5 μm (out of 110 μm) in the middle of the overall picture to minimise any potential effects of chromatic aberrations. The system vibration and drift were measured for up to 1 min using ∼500 nm emission 0.03 μm latex beads (L5155, Sigma-Aldrich) deposited on a glass cover slip and was found to be typically within ±2 nm (Fig. S5†).

For the scanning confocal microscopy Leica TCS SP2 Inverted Laser Scanning Confocal Microscope, equipped with a 488 nm Ar Laser (125 mW) and two PMT detectors, was used. Images were taken under HCX PL APO 63X/1.40 (oil) objective. Scanning formats were selected according to priority of image in terms of quality or data acquisition time, high (*e.g.*, 4096-pixel) or small (*e.g.*, 256-pixel), and Line : Frame averages were taken (*e.g.*, 2 : 3, *i.e.*, 2-line-average and 3-frame-average were taken for the image of one channel) or not (*e.g.*, 1 : 1). The emission range of each channel was adjusted according to the FWHM of each QD type, and different colours were assigned to each QD to differentiate one from the other. The parameters were saved for each channel, and then the channels were added to sequential scan mode in Leica Confocal Software (v2.1), which provides images to be taken in series. The pixel sizes depended on the image size and scan format and were calculated by dividing the size of the scan field by the size of the scan format. For instance, the scan field is 10.17 μm under 63X objective with a zoom factor of 23.4, and if scan format of 256 × 256 is selected, the pixel size will be (10.17 μm/256 = 0.03972 μm) about 40 nm. As far as the data acquisition time of the image is concerned, it is calculated by dividing the size of the scan format by the scan rate, which was set to 400 Hz. If there is no line or frame average taken, the data acquisition time will be (256-pixel/2 × 400 Hz = 0.32 seconds) 320 milliseconds.

### QDOSS analysis

The peak emission wavelength and width of QDs in this work were selected to match the parameters of the systems and, in particular, those of the Kurios-WB1 liquid crystal filter. This allowed us to alleviate the spectral channel cross talk (see Fig. S6† for the corresponding data). Detailed analysis based on the Kurious filter data The DNA origami data collection time was between 0.02 s and 0.05 s per frame and the overall collection time never exceeded 1 s. Images from each spectral channel were loaded into ImageJ and a stack was generated. In each case the stacks were aligned by the sides of the identical image frames. The individual images in a stack data were analysed using the ImageJ image manipulation software and GDSC SMLM following the methodology described below. The PSF of the microscope was approximated to a 2D Gaussian function using the PSF Calculator tool, and the initial parameters for the 2D Gaussian were set to the initial standard deviation value in pixels. The CCD gain was calculated by the mean-variance test using a set of calibration flat field exposures of a white card collected using the custom-made system and given as Analogue-to-Digital Units (ADUs) per photon in each image. 2D Gaussian fitting based on the Levenberg–Marquardt method was used in the reconstruction. All the candidate maxima of the interested region were found in the “Peak Fit” tool. Following the analysis described above, the images were scaled up by a factor of 10 for the final overlay to produce a reconstructed image. The quantification of the image resolution was calculated using the standard expression.^51^

## Conflicts of interest

The authors declare there are no conflicts of interest.

## Supplementary Material

RA-012-D2RA04316E-s001
