# Supplementary material for: Engineering a DNA origami mediated multicolour quantum dot platform for sub-diffraction spectral separation imaging
Source: RSC Adv. 2022 Aug 22;12(37):23778–85. doi: 10.1039/d2ra04316e (PMC9394590; doi:10.1039/d2ra04316e)
Supplement: RA-012-D2RA04316E-s001 [file RA-012-D2RA04316E-s001.pdf]

Electronic Supplementary Material (ESI) for RSC Advances.
This journal is © The Royal Society of Chemistry 2022

Supplementary Information

Engineering a DNA Origami Mediated Multicolour Quantum Dots Platform for Sub-diffraction Spectral Separation Imaging

Da Huang,[a] Lucy Haddad,[b] Fahmida Rahman,[b] Matteo Palma,*[a] and Andrei Sapelkin*[b]

a Department of Chemistry, Queen Mary University of London, London E1 4NS, United Kingdom
b Department of Physics and Astronomy, Queen Mary University of London, London E1 4NS, United Kingdom

DNA Origami Standard

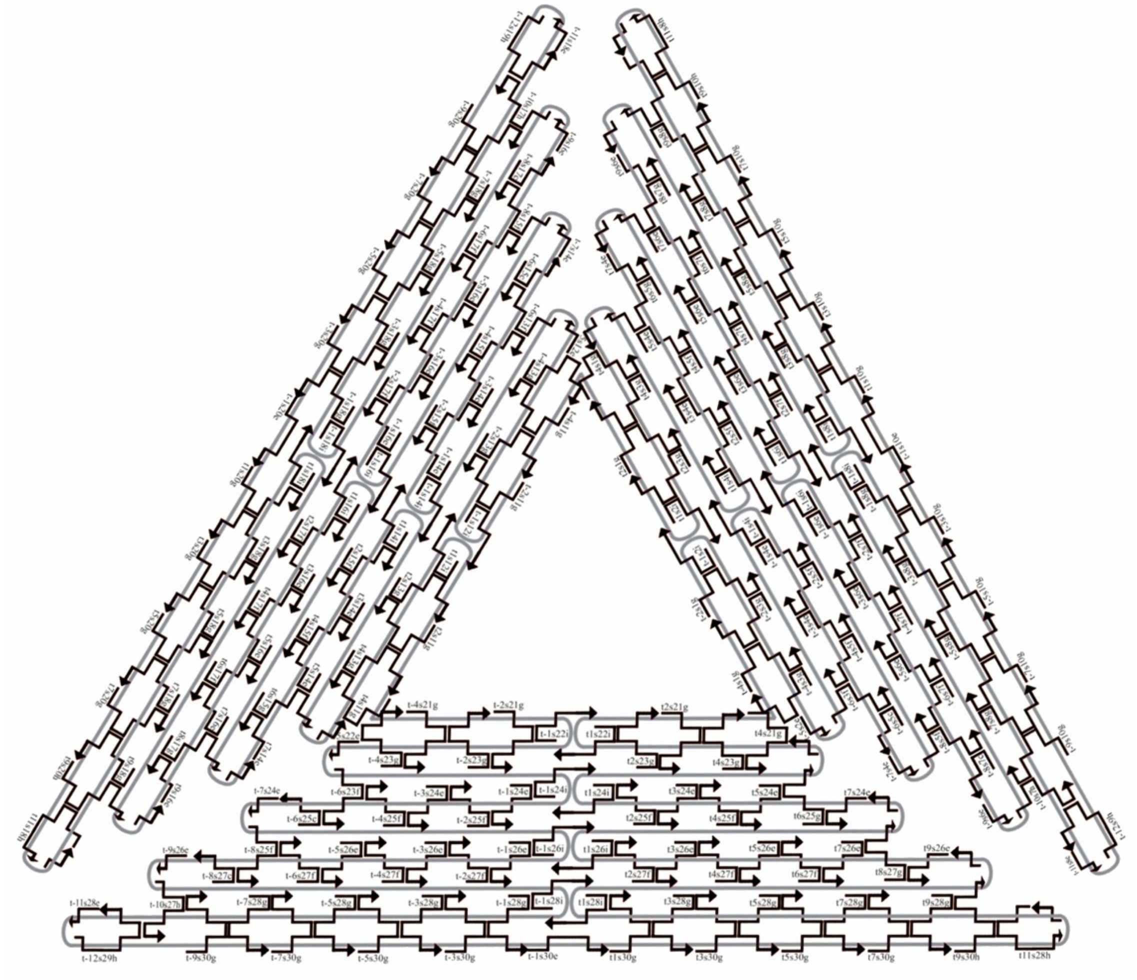

Fig. S1 Illustration and strands map of triangular DNA origami.

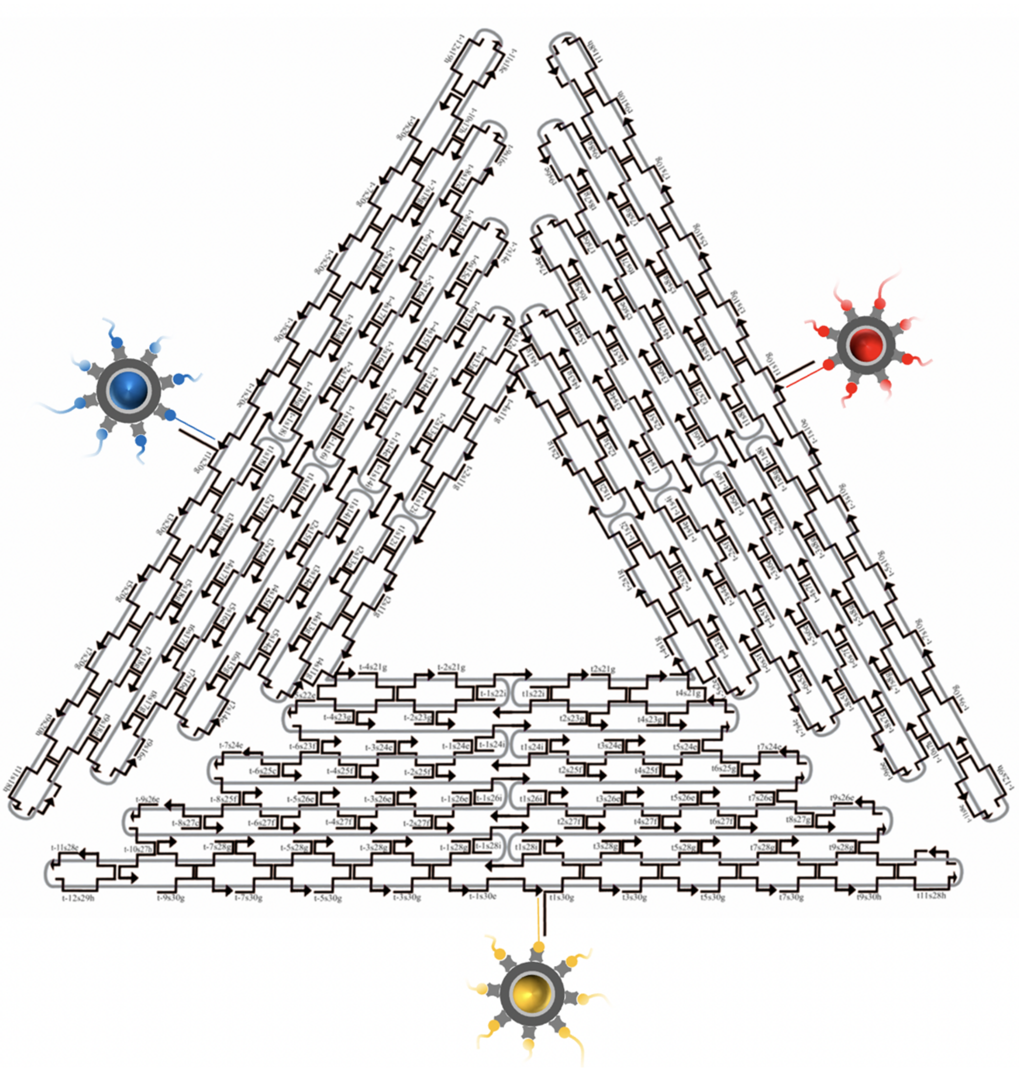

Fig. S2 Quantum Dots modifications on triangular DNA origami.

Table S1 List of modified staple strands

Oligo Name	Sequence (5'-3')
Sticky End t1s10g	AGAGAATAACATAAAAACAGGGAAGCGCATTATGTGGTGGTG
Sticky End t1s20g	ACAGGTAGAAAGATTCATCAGTTGAGATTTAGGCATGGGCGG
Sticky End t1s30g	CGAGAAAGGAAGGGAAGCGTACTATGGTTGCTAGCTTTGAGG
A37-ext'	Biotin-AAAAACACCACCACA
B37-ext'	Biotin-AAAAACCGCCCATGC
C37-ext'	Biotin-AAAAACCTCAAAGCT

Table S2 List of unmodified staple strands

Oligo Name	Sequence (5'-3')
t11s18h	AATACTGCGGAATCGTAGGGGGTAATAGTAAAATGTTTAGACT
t11s28h	TCTTTGATTAGTAATAGTCTGTCCATCACGCAAATTAACCGTT
t11s8h	CAGAAGGAAACCGAGGTTTTTAAGAAAAGTAAGCAGATAGCCG
t1s12i	TCATATGTGTAATCGTAAAACTAGTCATTTTC
t1s14i	GTGAGAAAATGTGTAGGTAAAGATACAACTTT
t1s16i	GGCATCAAATTTGGGGCGCGAGCTAGTTAAAG
t1s18i	TTCGAGCTAAGACTTCAAATATCGGGAACGAG
t1s22i	TCGGGAGATATACAGTAACAGTACAAATAATT
t1s24i	CCTGATTAAAGGAGCGGAATTATCTCGGCCTC
t1s26i	GCAAATCACCTCAATCAATATCTGCAGGTCGA
t1s28i	CGACCAGTACATTGGCAGATTCACCTGATTGC
t1s2i	CGGGGTTTCCTCAAGAGAAGGATTTTGAATTA
t1s4i	AGCGTCATGTCTCTGAATTTACCGACTACCTT
t1s6i	TTCATAATCCCCTTATTAGCGTTTTTCTTACC
t1s8i	ATGGTTTATGTCACAATCAATAGATATTAAAC
t2s11g	AGAAAAGCCCCAAAAAGAGTCTGGAGCAAACAATCACCAT
t2s13g	ACAGTCAAAGAGAATCGATGAACGACCCCGGTTGATAATC
t2s15f	ATAGTAGTATGCAATGCCTGAGTAGGCCGGAG
t2s17f	AACCAGACGTTTAGCTATATTTTCTTCTACTA
t2s1g	GATAAGTGCCGTCGAGCTGAAACATGAAAGTATACAGGAG
t2s21g	CCTGATTGCTTTGAATTGCGTAGATTTTCAGGCATCAATA
t2s23g	TGGCAATTTTTAACGTCAGATGAAAACAATAACGGATTCG
t2s25f	AAGGAATTACAAAGAAACCACCAGTCAGATGA
t2s27f	GGACATTCACCTCAAATATCAAACACAGTTGA
t2s3g	TTTGATGATTAAGAGGCTGAGACTTGCTCAGTACCAGGCG
t2s5f	CCGGAACCCAGAATGGAAAGCGCAACATGGCT
t2s7f	AAAGACAACATTTTCGGTCATAGCCAAAATCA
t3s10g	GTCAGAGGGTAATTGATGGCAACATATAAAAGCGATTGAG
t3s14e	CAATATGACCCTCATATATTTTAAAGCATTAA
t3s16e	CATCCAATAAATGGTCAATAACCTCGGAAGCA
t3s18g	AACTCCAAGATTGCATCAAAAAGATAATGCAGATACATAA
t3s20g	CGCCAAAAGGAATTACAGTCAGAAGCAAAGCGCAGGTCAG
t3s24e	TAATCCTGATTATCATTTTGCGGAGAGGAAGG
t3s26e	TTATCTAAAGCATCACCTTGCTGATGGCCAAC
t3s28g	AGAGATAGTTTGACGCTCAATCGTACGTGCTTTCCTCGTT
t3s30g	AGAATCAGAGCGGGAGATGGAAATACCTACATAACCCTTC
t3s4e	TGTACTGGAAATCCTCATTAAAGCAGAGCCAC
t3s6e	CACCGGAAAGCGCGTTTTCATCGGAAGGGCGA
t3s8g	CATTCAACAAACGCAAAGACACCAGAACACCCTGAACAAA
t4s11g	GCAAATATTTAAATTGAGATCTACAAAGGCTACTGATAAA
t4s13g	CGTTCTAGTCAGGTCATTGCCTGACAGGAAGATTGTATAA
t4s15f	CAGGCAAGATAAAAATTTTTAGAATATTCAAC
t4s17f	GATTAGAGATTAGATACATTTCGCAAATCATA

t4s1g	TAGCCCGGAATAGGTGAATGCCCCCTGCCTATGGTCAGTG
t4s21g	GCGCAGAGGCGAATTAATTATTTGCACGTAAATTCTGAAT
t4s23g	GATTATACACAGAAATAAAGAAATACCAAGTTACAAAATC
t4s25f	TAGGAGCATAAAAGTTTGAGTAACATTGTTTG
t4s27f	TGACCTGACAAATGAAAAATCTAAAATATCTT
t4s3g	TTTAACGGTTCGGAACCTATTATTAGGGTTGATATAAGTA
t4s5f	CTCAGAGCATATTCACAAACAAATTAATAAGT
t4s7f	GGAGGGAATTTAGCGTCAGACTGTCCGCCTCC
t5s10g	GATAACCCACAAGAATGTTAGCAAACGTAGAAAATTATTC
t5s14e	TTAATGCCTTATTTCAACGCAAGGGCAAAGAA
t5s16e	TTAGCAAATAGATTTAGTTTGACCAGTACCTT
t5s18g	TAATTGCTTTACCCTGACTATTATGAGGCATAGTAAGAGC
t5s20g	AACACTATCATAACCCATCAAAAATCAGGTCTCCTTTTGA
t5s24e	AATGGAAGCGAACGTTATTAATTTCTAACAAC
t5s26e	TAATAGATCGCTGAGAGCCAGCAGAAGCGTAA
t5s28g	GAATACGTAACAGGAAAAACGCTCCTAAACAGGAGGCCGA
t5s30g	TTAAAGGGATTTTAGATACCGCCAGCCATTGCGGCACAGA
t5s4e	CCTTGAGTCAGACGATTGGCCTTGCGCCACCC
t5s6e	TCAGAACCCAGAATCAAGTTTGCCGGTAAATA
t5s8g	TTGACGGAAATACATACATAAAGGGCGCTAATATCAGAGA
t6s15g	ATAAAGCCTTTGCGGGAGAAGCCTGGAGAGGGTAG
t6s17f	TAAGAGGTCAATTCTGCGAACGAGATTAAGCA
t6s25g	TCAATAGATATTAAATCCTTTGCCGGTTAGAACCT
t6s27f	CAATATTTGCCTGCAACAGTGCCATAGAGCCG
t6s5g	CAGAGCCAGGAGGTTGAGGCAGGTAACAGTGCCCG
t6s7f	ATTAAAGGCCGTAATCAGTAGCGAGCCACCCT
t7s10g	ATAAGAGCAAGAAACATGGCATGATTAAGACTCCGACTTG
t7s14e	ATGACCCTGTAATACTTCAGAGCA
t7s16e	TAAAGCTATATAACAGTTGATTCCCATTTTTG
t7s18g	CGGATGGCACGAGAATGACCATAATCGTTTACCAGACGAC
t7s20g	GATAAAAACCAAAATATTAAACAGTTCAGAAATTAGAGCT
t7s24e	ACAATTCGACAACTCGTAATACAT
t7s26e	TTGAGGATGGTCAGTATTAACACCTTGAATGG
t7s28g	CTATTAGTATATCCAGAACAATATCAGGAACGGTACGCCA
t7s30g	GAATCCTGAGAAGTGTATCGGCCTTGCTGGTACTTTAATG
t7s4e	GCCGCCAGCATTGACACCACCCTC
t7s6e	AGAGCCGCACCATCGATAGCAGCATGAATTAT
t7s8g	CACCGTCACCTTATTACGCAGTATTGAGTTAAGCCCAATA
t8s17g	TAATTGCTTGGAAGTTTCATTCCAAATCGGTTGTA
t8s27g	CGCGAACTAAAACAGAGGTGAGGCTTAGAAGTATT
t8s7g	AGCCATTTAAACGTCACCAATGAACACCAGAACCA
t9s10h	TATCTTACCGAAGCCCAAACGCAATAATAACGAAAATCACCAG
t9s16e	ACTAAAGTACGGTGTCGAATATAA
t9s18g	TGCTGTAGATCCCCCTCAAATGCTGCGAGAGGCTTTTGCA
t9s20h	AAAGAAGTTTTGCCAGCATAAATATTCATTGACTCAACATGTT
t9s26e	ACCACCAGCAGAAGATGATAGCCC

t9s28g	TAAAACATTAGAAGAACTCAAACTTTTTATAATCAGTGAG
t9s30h	GCCACCGAGTAAAAGAACATCACTTGCCTGAGCGCCATTAAAA
t9s6e	CCATTAGCAAGGCCGGGGGAATTA
t9s8g	GAGCCAGCGAATACCCAAAAGAACATGAAATAGCAATAGC
t10s17h	ACCAACCTAAAAAATCAACGTAACAAATAAATTGGGCTTGAGA
t10s27h	AACTCACATTATTGAGTGTTGTTCCAGAAACCGTCTATCAGGG
t10s7h	ACGACAATAAATCCCGACTTGCGGGAGATCCTGAATCTTACCA
t12s19h	CCTGACGAGAAACACCAGAACGAGTAGGCTGCTCATTCAGTGA
t12s29h	ACGTGGACTCCAACGTCAAAGGGCGAATTTGGAACAAGAGTCC
t12s9h	TGCTATTTTGCACCCAGCTACAATTTTGTTTTGAAGCCTTAAA
t1s10e	AGAGAATAACATAAAAACAGGGAAGCGCATTA
t1s12i	AGGGATAGCTCAGAGCCACCACCCCATGTCAA
t1s14e	ATTTTCTGTCAGCGGAGTGAGAATACCGATAT
t1s14i	CAACAGTTTATGGGATTTTGCTAATCAAAAGG
t1s16e	ATTCGGTCTGCGGGATCGTCACCCGAAATCCG
t1s16i	GCCGCTTTGCTGAGGCTTGCAGGGGAAAAGGT
t1s18g	CGACCTGCGGTCAATCATAAGGGAACGGAACAACATTATT
t1s18i	GCGCAGACTCCATGTTACTTAGCCCGTTTTAA
t1s20e	ACAGGTAGAAAGATTCATCAGTTGAGATTTAG
t1s22i	CGCGTCTGATAGGAACGCCATCAACTTTTACA
t1s24e	CAGTTTGACGCACTCCAGCCAGCTAAACGACG
t1s24i	AGGAAGATGGGGACGACGACAGTAATCATATT
t1s26e	GCCAGTGCGATCCCCGGGTACCGAGTTTTTCT
t1s26i	CTCTAGAGCAAGCTTGCATGCCTGGTCAGTTG
t1s28g	TTTCACCAGCCTGGCCCTGAGAGAAAGCCGGCGAACGTGG
t1s28i	CCTTCACCGTGAGACGGGCAACAGCAGTCACA
t1s2i	CCTTTTTTCATTTAACAATTTCATAGGATTAG
t1s30e	CGAGAAAGGAAGGGAAGCGTACTATGGTTGCT
t1s4e	TTATCAAACCGGCTTAGGTTGGGTAAGCCTGT
t1s4i	TTTAACCTATCATAGGTCTGAGAGTTCCAGTA
t1s6e	TTAGTATCGCCAACGCTCAACAGTCGGCTGTC
t1s6i	AGTATAAAATATGCGTTATACAAAGCCATCTT
t1s8g	TTTCCTTAGCACTCATCGAGAACAATAGCAGCCTTTACAG
t1s8i	CAAGTACCTCATTCCAAGAACGGGAAATTCAT
t2s11g	CCTCAGAACCGCCACCCAAGCCCAATAGGAACGTAAATGA
t2s13g	AGACGTTACCATGTACCGTAACACCCCTCAGAACCGCCAC
t2s15f	CACGCATAAGAAAGGAACAACTAAGTCTTTCC
t2s17f	ATTGTGTCTCAGCAGCGAAAGACACCATCGCC
t2s1g	AAAACAAAATTAATTAAATGGAAACAGTACATTAGTGAAT
t2s21g	GCTCATTTTTTAACCAGCCTTCCTGTAGCCAGGCATCTGC
t2s23g	GTAACCGTCTTTCATCAACATTAAAATTTTTGTTAAATCA
t2s25f	ACGTTGTATTCCGGCACCGCTTCTGGCGCATC
t2s27f	CCAGGGTGGCTCGAATTCGTAATCCAGTCACG
t2s3g	AGAGTCAAAAATCAATATATGTGATGAAACAAACATCAAG
t2s5f	ACTAGAAATATATAACTATATGTACGCTGAGA
t2s7f	TCAATAATAGGGCTTAATTGAGAATCATAATT

t3s10g	AACGTCAAAAATGAAAAGCAAGCCGTTTTTATGAAACCAA
t3s14e	GTTTTGTCAGGAATTGCGAATAATCCGACAAT
t3s16e	GACAACAAGCATCGGAACGAGGGTGAGATTTG
t3s18g	TATCATCGTTGAAAGAGGACAGATGGAAGAAAAATCTACG
t3s20g	TTAATAAAACGAACTAACCGAACTGACCAACTCCTGATAA
t3s24e	TGTAGATGGGTGCCGGAAACCAGGAACGCCAG
t3s26e	GGTTTTCCATGGTCATAGCTGTTTGAGAGGCG
t3s28g	GTTTGCGTCACGCTGGTTTGCCCCAAGGGAGCCCCCGATT
t3s30g	TAGAGCTTGACGGGGAGTTGCAGCAAGCGGTCATTGGGCG
t3s4e	GATTAAGAAATGCTGATGCAAATCAGAATAAA
t3s6e	CACCGGAATCGCCATATTTAACAAAATTTACG
t3s8g	AGCATGTATTTCATCGTAGGAATCAAACGATTTTTTGTTT
t4s11g	AGGTTTAGTACCGCCATGAGTTTCGTCACCAGGATCTAAA
t4s13g	AGCGTAACTACAAACTACAACGCCTATCACCGTACTCAGG
t4s15f	TAGTTGCGAATTTTTTCACGTTGATCATAGTT
t4s17f	GTACAACGAGCAACGGCTACAGAGGATACCGA
t4s1g	GAGCAAAAGAAGATGAGTGAATAACCTTGCTTATAGCTTA
t4s21g	GTTAAAATTCGCATTAATGTGAGCGAGTAACACACGTTGG
t4s23g	GGATAGGTACCCGTCGGATTCTCCTAAACGTTAATATTTT
t4s25f	AGTTGGGTCAAAGCGCCATTCGCCCCGTAATG
t4s27f	CGCGCGGGCCTGTGTGAAATTGTTGGCGATTA
t4s3g	ACATAGCGCTGTAAATCGTCGCTATTCATTTCAATTACCT
t4s5f	GTTAAATACAATCGCAAGACAAAGCCTTGAAA
t4s7f	CCCATCCTCGCCAACATGTAATTTAATAAGGC
t5s10g	TCCCAATCCAAATAAGATTACCGCGCCCAATAAATAATAT
t5s16e	AACAGCTTGCTTTGAGGACTAAAGCGATTATA
t5s18g	CCAAGCGCAGGCGCATAGGCTGGCAGAACTGGCTCATTAT
t5s20g	ACCAGTCAGGACGTTGGAACGGTGTACAGACCGAAACAAA
t5s26e	TGCTGCAAATCCGCTCACAATTCCCAGCTGCA
t5s28g	TTAATGAAGTTTGATGGTGGTTCCGAGGTGCCGTAAAGCA
t5s30g	CTAAATCGGAACCCTAAGCAGGCGAAAATCCTTCGGCCAA
t5s6e	GTGTGATAAGGCAGAGGCATTTTCAGTCCTGA
t5s8g	ACAAGAAAGCAAGCAAATCAGATAACAGCCATATTATTTA
t6s13f	ACAGACAGCCCAAATCTCCAAAAAAAAATTTCTTA
t6s15c	CGAGGTGAGGCTCCAAAAGGAGCC
t6s17f	ACCCCCAGACTTTTTCATGAGGAACTTGCTTT
t6s23f	CGGCGGATTGAATTCAGGCTGCGCAACGGGGGATG
t6s25c	TGGCGAAATGTTGGGAAGGGCGAT
t6s27f	TGTCGTGCACACAACATACGAGCCACGCCAGC
t6s3f	TCCCTTAGAATAACGCGAGAAAACTTTTACCGACC
t6s5c	GTTTGAAATTCAAATATATTTTAG
t6s7f	AATAGATAGAGCCAGTAATAAGAGATTTAATG
t7s10g	GCCAGTTACAAAATAATAGAAGGCTTATCCGGTTATCAAC
t7s18g	AAAACACTTAATCTTGACAAGAACTTAATCATTGTGAATT
t7s20g	ACCTTATGCGATTTTATGACCTTCATCAAGAGCATCTTTG
t7s28g	TTCCAGTCCTTATAAATCAAAAGAGAACCATCACCCAAAT

t7s30g	CAAGTTTTTTGGGGTCGAAATCGGCAAAATCCGGGAAACC
t7s8g	GCGCCTGTTATTCTAAGAACGCGATTCCAGAGCCTAATTT
t8s15f	CGGTTTATCAGGTTTCCATTAAACGGGAATACACT
t8s17c	GGCAAAAGTAAAATACGTAATGCC
t8s25f	TCTTCGCTATTGGAAGCATAAAGTGTATGCCCGCT
t8s27c	GCGCTCACAAGCCTGGGGTGCCTA
t8s5f	TTCTGACCTAAAATATAAAGTACCGACTGCAGAAC
t8s7c	TCAGCTAAAAAAGGTAAAGTAATT
t9s10g	ACGCTAACGAGCGTCTGGCGTTTTAGCGAACCCAACATGT
t9s20g	TGGTTTAATTTCAACTCGGATATTCATTACCCACGAAAGA
t9s30g	CGATGGCCCACTACGTATAGCCCGAGATAGGGATTGCGTT
tsrem1	GCGCTTAATGCGCCGCTACAGGGC
t5s2et6s23c3T	TTAATTAATTTTTTACCATATCAAA
t7s4et8s25c2T	TTAATTTCATCTTAGACTTTACAA
t9s6et10s27c1T	CTGTCCAGACGTATACCGAACGA
t11s8et12s29c0T	TCAAGATTAGTGTAGCAATACT
t5s12et6s3c3T	TGTAGCATTCCTTTTATAAACAGTT
t7s14et8s5c2T	TTTAATTGTATTTCCACCAGAGCC
t9s16et10s7c1T	ACTACGAAGGCTTAGCACCATTA
t11s18et12s9c0T	ATAAGGCTTGCAACAAAGTTAC
t5s22et6s13c-T	GTGGGAACAAATTTCTATTTTTGAG
t7s24et8s15c2T	CGGTGCGGGCCTTCCAAAAACATT
t9s26et10s17c1T	ATGAGTGAGCTTTTAAATATGCA
t11s28et12s19c0T	ACTATTAAAGAGGATAGCGTCC

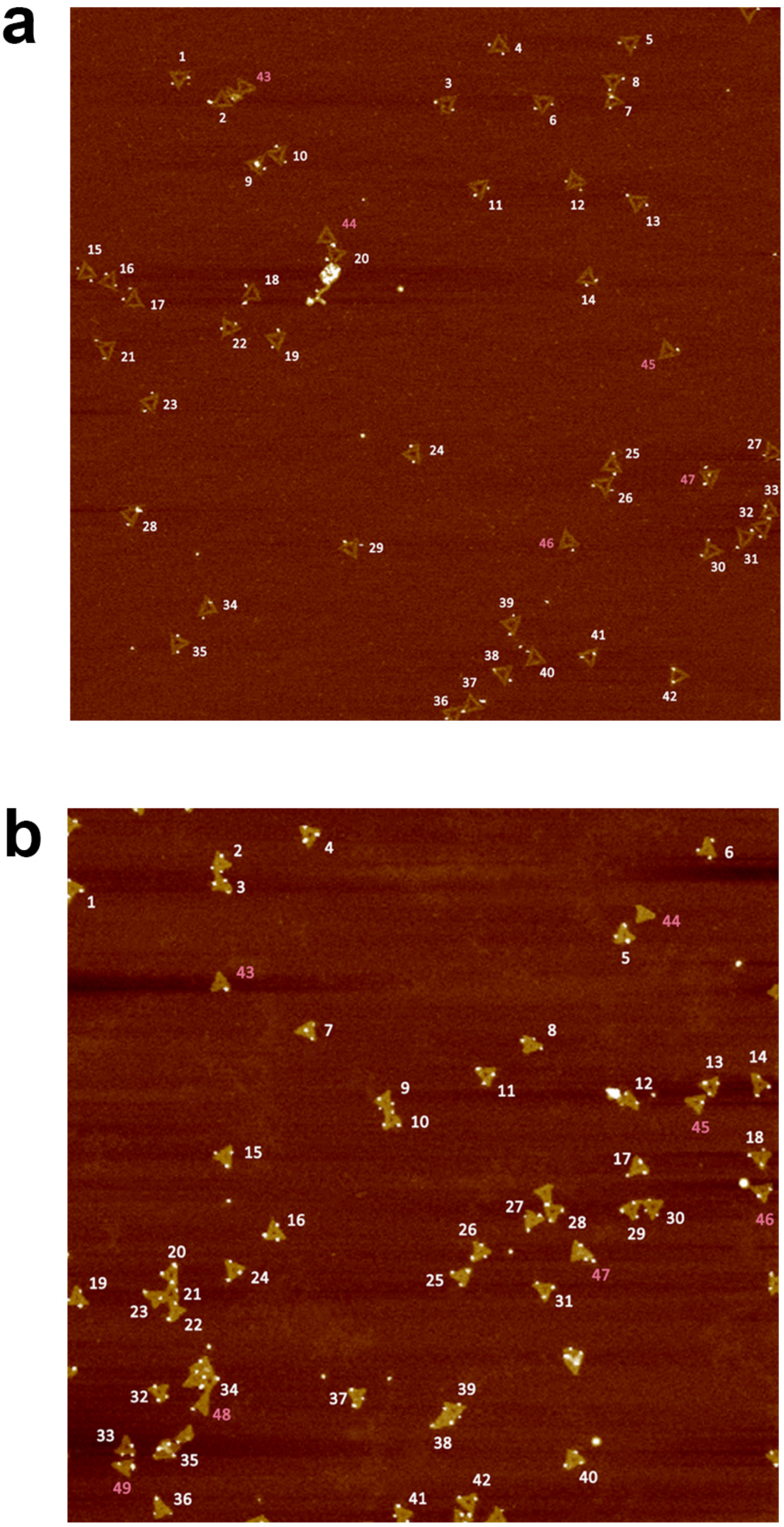

Fig. S3 Yielding Statistics of QD(s) modified DNA origami. a) Sample of counting for two QDs modified DNA origami, yielding 89%; b) Sample of three QDs modified DNA origami, yielding 86%. The cross-section was measured and analysed as QD spacing distribution in the following tables.

Table S3 Analysis QD spacing on 2-QDs-bound DNA origami

QD Spacing (nm)	Probability (%)
30 ±5	0.0
40 ±5	1.1
50 ±5	6.5
60 ±5	24.8
70 ±5	37.6
80 ±5	21.5
90 ±5	7.9
100 ±5	0.6
110 ±5	0.0

Table S4 Analysis QD spacing on 3-QDs-bound DNA origami

QD Spacing (nm)	Probability (%)
30 ±5	0
40 ±5	0.8
50 ±5	8.5
60 ±5	31.8
70 ±5	37.7
80 ±5	17.3
90 ±5	3.2
100 ±5	0.7
110 ±5	0.0

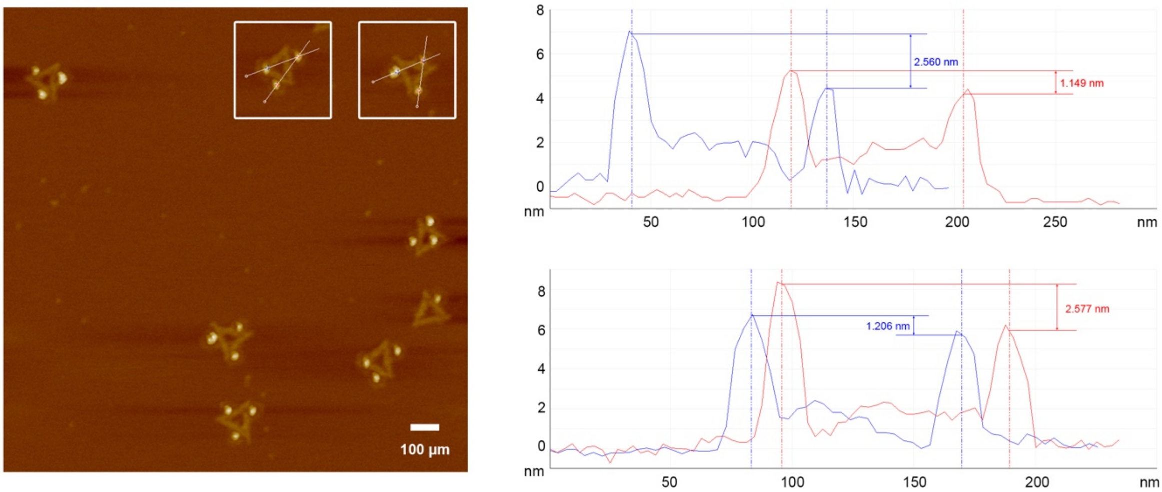

Fig. S4 AFM topographical images and corresponding height profiles of the QDs attached to DNA origami, demonstrating the difference between the height (and hence the size of QDs). Height profiles show that all QDs on each DNA-origami are of different size and that we achieved targeted attachment of three different QDs on the same DNA origami.

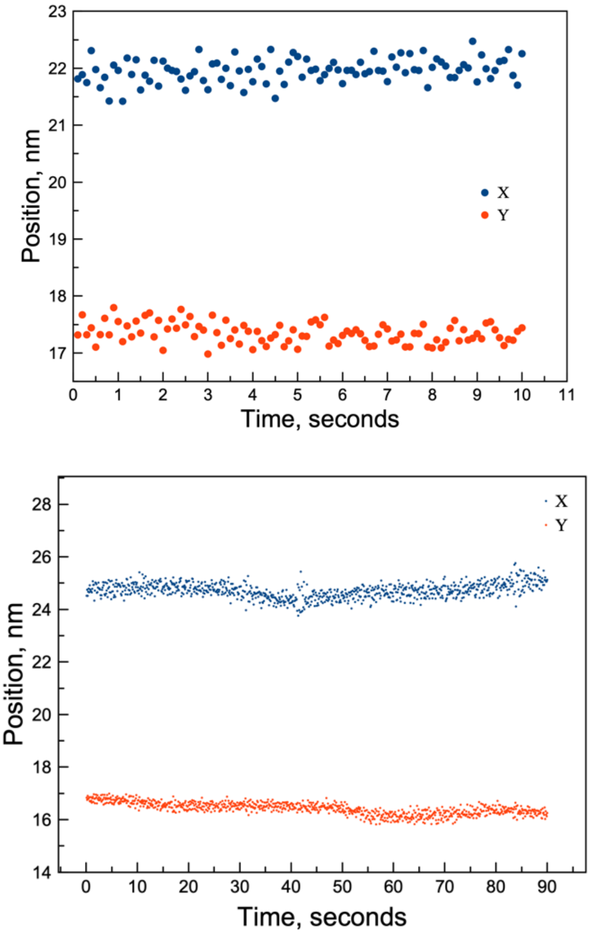

Fig. S5 Quantum Dot-Based Optical Spectral Separation (QDOSS) Microscopy drift analysis for the positioning system.

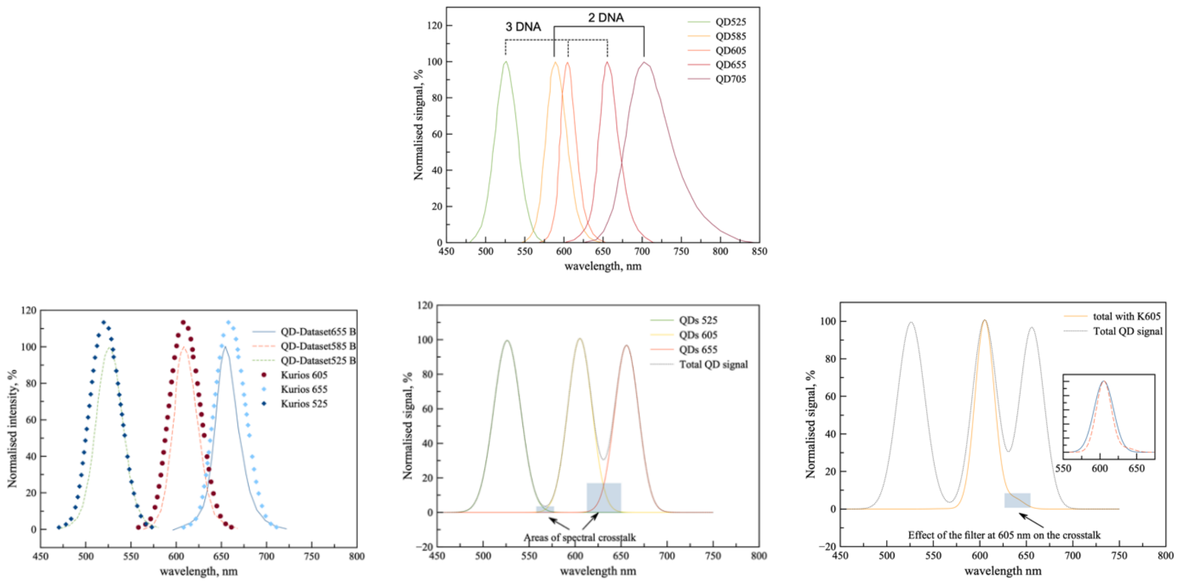

Fig. S6 Top row: Emission spectra of quantum dots used in this study. Bottom row: matching between the Kurios WB-1 filter and emission profiles for 3 DNA labelling; spectral crosstalk and effect of the filter centred at 605 nm on the corresponding QD emission peak. Inset shows the difference between the original emission at 605 nm (solid line) and that after the filter is applied to the total signal from all 3 QDs: the difference illustrates the level of the crosstalk between spectral channels.

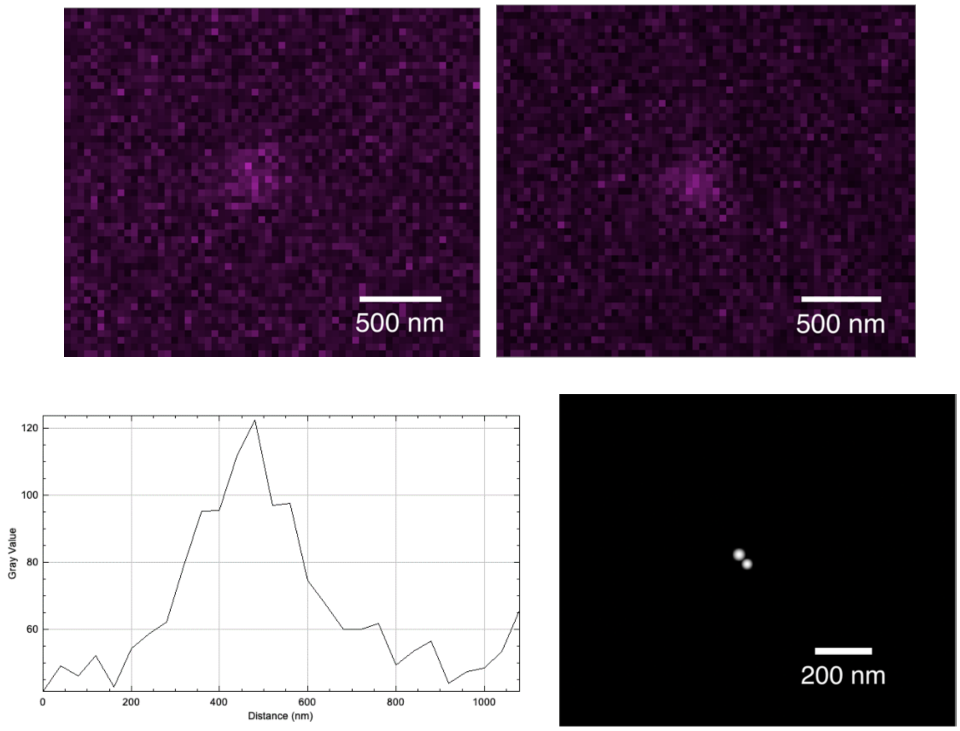

Fig. S7 Top row: Diffraction limited 2 DNA images collected at 585 and 705 nm. Bottom row: the corresponding intensity profile for one of the images illustrating the signal-to-noise ratio and super-resolution (following spectral separation and data analysis) image of triangular DNA origami with two colour QDs. Please see also see attached numerical analysis data tables.

Table S5. GDSC SMLM Peak fitting plugin output data for 2 DNA system above.

Source	Frame	origX	origY	origValue	Error	Noise	SNR	Background	+/-	Signal	+/-	X	+/-	Y	+/-	Precision (nm)
Stack:	1	28	27	146	0.408584	4.03546	213.405	9.07881	0.0143371	861.186	2.86451	1173.21	0.465761	1061.77	0.465779	9.95043
Stack:	2	27	25	95	0.320312	4.3733	180.731	11.3236	0.014343	790.391	2.86569	1122.6	0.507444	1002.68	0.507435	11.3625

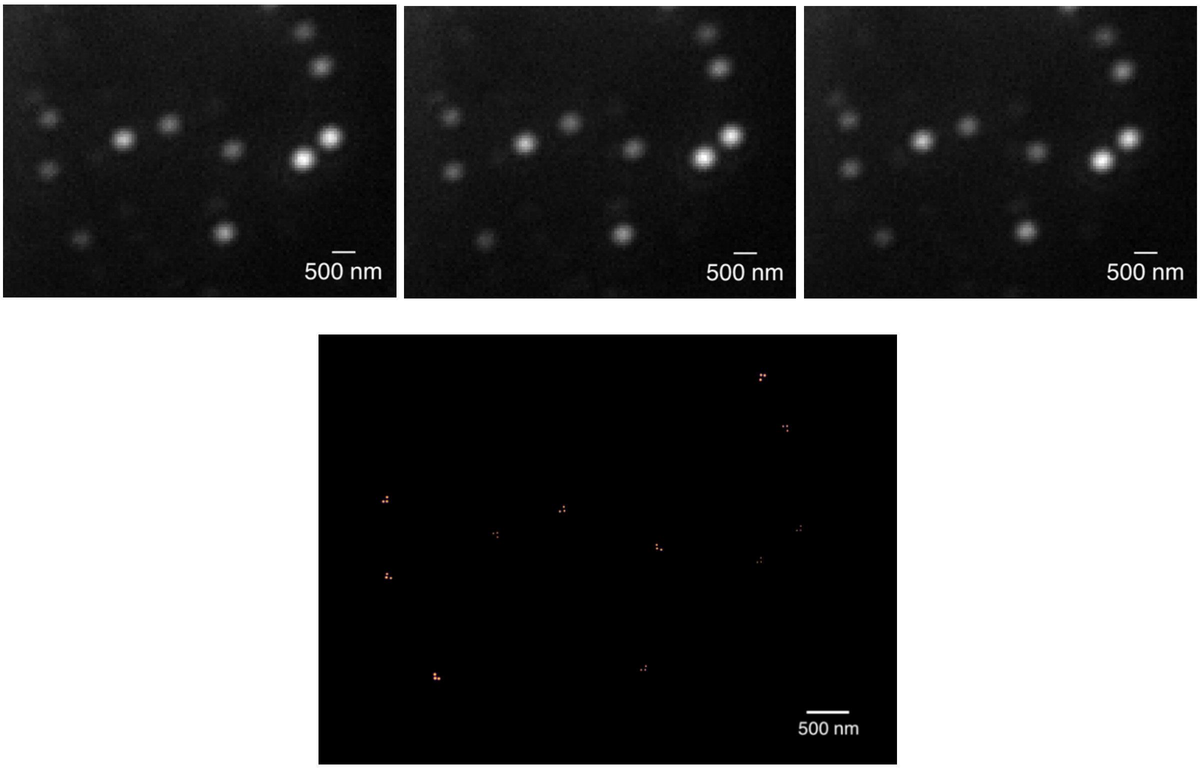

Fig. S8 Diffraction limited (top row) and the corresponding super-resolution (following spectral separation and data analysis) images of triangular DNA origami with three colour (525, 606 and 655 nm) QDs. Please also see attached numerical analysis data tables.

Table S6. GDSC SMLM Peak fitting plugin output data for 3 DNA system above.

Source	Frame	origX	origY	origValue	Error	Noise	SNR	Background	+/-	Signal	+/-	X	+/-	Y	+/-	Precision (nm)
Stack:	3	107	54	1963	0.966065	4.48856	3985.86	434.016	0.0280842	17890.8	5.18112	6905.06	0.0640306	3543.63	0.0639007	2.0473
Stack:	3	116	47	1912	0.968024	4.48856	3667.13	431.439	0.0279078	16460.1	5.1678	7523.07	0.0696649	3049.13	0.0695157	2.14588
Stack:	3	43	47	1728	0.929255	4.48856	2583.62	442.227	0.025246	11596.7	5.04408	2787.29	0.0981505	3089.97	0.0981515	2.627
Stack:	3	79	81	1612	0.922469	4.48856	2110.32	434.305	0.0252446	9472.31	5.04379	5097.58	0.120166	5228.96	0.120165	2.9645
Stack:	3	113	22	1528	0.913142	4.48856	1751.82	441.148	0.0252464	7863.16	5.04417	7312.73	0.144753	1423.77	0.144756	3.32316
Stack:	3	59	42	1471	0.920812	4.48856	1455.49	445.586	0.0252481	6533.03	5.0445	3834.8	0.174224	2747.08	0.174224	3.73506
Stack:	3	81	51	1425	0.916522	4.48856	1406.23	441.653	0.0252466	6311.95	5.0442	5280.65	0.18033	3334.86	0.180328	3.81844
Stack:	3	16	39	1368	0.721167	4.48856	941.381	453.513	0.0252425	4225.44	5.04338	1063.42	0.269372	2598.42	0.269396	4.98575
Stack:	3	106	9	1361	0.774546	4.48856	1010.74	449.632	0.025245	4536.75	5.04387	6905.54	0.250898	625.866	0.250889	4.74942
Stack:	2	107	53	1979	0.964643	4.45067	3924.41	434.803	0.0280659	17466.3	5.1818	6903.19	0.0655538	3480.2	0.0654275	2.07294
Stack:	1	106	54	2007	0.961768	4.49544	4081.82	434.059	0.0280478	18349.6	5.17923	6848.76	0.0624245	3549.71	0.0623003	2.01878
Stack:	3	16	58	1325	0.845301	4.48856	887.122	447.604	0.0252477	3981.9	5.04441	1054.99	0.285847	3786.28	0.285848	5.19445
Stack:	2	116	46	1943	0.966672	4.45067	3662.22	432.108	0.0279025	16299.3	5.16901	7526.11	0.0703213	2984.2	0.0701719	2.15542
Stack:	1	115	47	1909	0.966448	4.49544	3476.15	432.104	0.0279356	15626.8	5.16928	7464.6	0.0733835	3052.75	0.0732228	2.21063
Stack:	2	43	48	1779	0.93806	4.45067	2682.91	442.622	0.0252459	11940.7	5.04406	2790.38	0.0953225	3157.21	0.0953243	2.5784
Stack:	3	28	82	1262	0.853982	4.48856	706.843	438.376	0.0252437	3172.71	5.04363	1815.18	0.358758	5360.27	0.35877	6.1008
Stack:	1	42	48	1805	0.931149	4.49544	2756.09	442.957	0.0252459	12389.8	5.04406	2725.24	0.0918674	3099.93	0.091869	2.52758
Stack:	2	77	81	1566	0.923626	4.45067	2069.49	434.919	0.025244	9210.61	5.04368	5033.91	0.123581	5230.38	0.123579	3.00984
Stack:	1	79	80	1589	0.92208	4.49544	2124.62	434.672	0.0252463	9551.09	5.04414	5107.36	0.119172	5170.5	0.119173	2.95063
Stack:	1	113	23	1564	0.918996	4.49544	1858.22	440.622	0.0252474	8353.53	5.04437	7317.98	0.136255	1497.22	0.136257	3.20217
Stack:	2	112	22	1509	0.915424	4.45067	1817.95	440.843	0.025247	8091.08	5.04428	7252.42	0.140675	1427.73	0.140677	3.25823
Stack:	1	58	42	1443	0.919469	4.49544	1408.16	445.969	0.0252476	6330.32	5.0444	3765.18	0.179804	2756.2	0.179804	3.81298
Stack:	2	59	41	1453	0.932065	4.45067	1430	446.433	0.0252478	6364.45	5.04444	3825.88	0.17884	2683.99	0.178839	3.78907
Stack:	1	82	52	1399	0.912541	4.49544	1365.74	441.898	0.0252444	6139.62	5.04376	5347.34	0.185392	3353.42	0.185395	3.88891
Stack:	2	81	50	1435	0.916666	4.45067	1477.41	441.746	0.0252465	6575.48	5.04418	5275.53	0.173102	3278.35	0.173102	3.71093
Stack:	1	15	39	1355	0.704126	4.49544	970.004	455.306	0.0252419	4360.6	5.04325	1007.01	0.261024	2600.67	0.261049	4.88205
Stack:	2	17	58	1416	0.872729	4.45067	1188.54	446.043	0.025247	5289.8	5.04428	1124.63	0.215172	3796.47	0.215175	4.27317
Stack:	1	16	57	1400	0.876469	4.49544	1162.29	446.752	0.0252469	5224.99	5.04426	1064.3	0.21784	3733.31	0.217844	4.32213
Stack:	2	16	38	1354	0.762224	4.45067	973.37	453.346	0.0252427	4332.15	5.04341	1064.97	0.262737	2533.22	0.26276	4.88599
Stack:	1	106	10	1336	0.783521	4.49544	951.216	448.923	0.0252453	4276.14	5.04394	6897.24	0.266185	701.198	0.266183	4.94791
Stack:	2	107	9	1334	0.786136	4.45067	922.61	448.076	0.0252456	4106.23	5.04401	6962.07	0.277199	632.518	0.277195	5.0687
Stack:	1	27	81	1283	0.837693	4.49544	744.335	438.755	0.0252411	3346.12	5.04309	1810.91	0.340176	5300.18	0.340182	5.87579
Stack:	2	29	83	1263	0.86585	4.45067	793.137	437.701	0.0252454	3529.99	5.04397	1872.71	0.32245	5366.22	0.322445	5.6326
